# Surveillance of fluoroquinolones resistance in rifampicin-susceptible tuberculosis in eastern China with whole-genome sequencing-based approach

**DOI:** 10.3389/fmicb.2024.1413618

**Published:** 2024-07-10

**Authors:** Yang Che, Yewei Lu, Yelei Zhu, Tianfeng He, Xiangchen Li, Junli Gao, Junshun Gao, Xiaomeng Wang, Zhengwei Liu, Feng Tong

**Affiliations:** ^1^Institute of Tuberculosis Prevention and Control, Ningbo Municipal Center for Disease Control and Prevention, Ningbo, China; ^2^Key Laboratory of Precision Medicine in Diagnosis and Monitoring Research of Zhejiang Province, Hangzhou, China; ^3^The Institute of TB Control, Zhejiang Provincial Center for Disease Control and Prevention, Hangzhou, China

**Keywords:** fluoroquinolones resistance, drug resistance, whole-genome sequencing, rifampicin-susceptible tuberculosis, hetero-resistance

## Abstract

**Background:**

Leveraging well-established DNA-level drug resistance mechanisms, whole-genome sequencing (WGS) has emerged as a valuable methodology for predicting drug resistance. As the most effective second-line anti-tuberculosis (anti-TB) drugs, fluoroquinoloness (FQs) are generally used to treat multidrug-resistant tuberculosis (MDR-TB, defined as being resistant to resistant to rifampicin and isoniazid) or rifampicin-resistant tuberculosis (RR-TB). However, FQs are also commonly used in the management of other bacterial infections. There are few published data on the rates of FQs resistance among rifampicin-susceptible TB. The prevalence of FQs resistance among TB patients who are rifampicin-susceptible has not been studied in Zhejiang Province, China. The goal of this study was to provide a baseline characterization of the prevalence of FQs resistance, particularly among rifampicin-susceptible TB in Zhejiang Province, China.

**Methods:**

Based on WGS, we have investigated the prevalence of FQs resistance among rifampicin-susceptible TB in Zhejiang Province. All pulmonary TB patients with positive cultures who were identified in Zhejiang area during TB drug resistance surveillance from 2018 to 2019 have enrolled in this population-based retrospective study.

**Results:**

The rate of FQs resistance was 4.6% (32/698) among TB, 4.0% (27/676) among rifampicin-susceptible TB, and 22.7% (5/22) among RR-TB. According to WGS, strains that differ within 12 single-nucleotide polymorphisms (SNPs) were considered to be transmission of FQ-resistant strains. Specifically, 3.7% (1/27) of FQs resistance was caused by the transmission of FQs-resistant strains among the rifampicin-susceptible TB and 40.7% (11/27) of FQs resistance was identified as hetero-resistance.

**Conclusion:**

The prevalence of FQs resistance among TB patients who were rifampicin-susceptible was severe in Zhejiang. The emergence of FQs resistance in TB isolates that are rifampicin-susceptible was mainly caused by the selection of drug-resistant strains. In order to prevent the emergence of FQs resistance, the WGS-based surveillance system for TB should be urgently established, and clinical awareness of the responsible use of FQs for respiratory infections should be enhanced.

## Background

As the most critical second-line anti-tuberculosis drugs, fluoroquinolones (FQs) have been recommended as Group A agents for use in multidrug-resistant tuberculosis (MDR-TB, defined as being resistant to resistant to rifampicin and isoniazid) or rifampicin-resistant tuberculosis (RR-TB) regimens by the World Health Organization (WHO) (World Health Organization, 2020). WHO also recommended levofloxacin, a FQ antibiotic, in the treatment regimen of isoniazid-resistant tuberculosis (Hr-TB) (World Health Organization, 2022a). Moxifloxacin, a fourth-generation FQ antibiotic, is under consideration by WHO for inclusion in four-month regimens to treat drug-susceptible tuberculosis because of its pharmacokinetics and drug penetration into macrophages (Dorman et al., 2021; World Health Organization, 2022b). Thus, as the cornerstone of the regimens for MDR/RR/Hr-TB, FQs will be the essential drugs in the shorter regimen for drug-susceptible TB in the future. The prevalence of FQs resistance among RR-TB has been investigated in many studies because it can significantly reduce the risk of treatment failure or relapse and death in RR-TB patients (Miotto et al., 2017; Siddiqui et al., 2019). Nevertheless, few studies have investigated the prevalence of FQs resistance among rifampicin-susceptible TB, and the majority of these studies show that FQs resistance in rifampicin-susceptible TB is uncommon (Hu et al., 2011; Schwalb et al., 2021; Zhang et al., 2022).

As a group of broad-spectrum antibiotics, FQs are widely used to treat other bacterial infections, especially respiratory infections. Additionally, FQs have been widely utilized in healthcare institutions for the diagnostic treatment of patients with suspected TB and for the empirical treatment of TB patients without positive drug susceptibility testing (DST) results in several studies (Wang et al., 2006; Ho et al., 2014). We should pay more attention to the emergence of FQs resistance in rifampicin-susceptible *Mycobacterium tuberculosis* (Mtb) isolates in TB high-burden settings by the inappropriate use and the vital role of FQs in TB treatment.

Based on DNA sequencing platforms, whole-genome sequencing, which reconstructed the complete genome’s DNA sequence, can provide high-resolution genotyping and identification (Witney et al., 2016; Walker et al., 2017; Cohen et al., 2019). FQs resistance in Mtb has been mainly attributed to mutations in the *gyrA* and *gyrB* genes, which code for two subunits of DNA gyrase (Ruiz, 2003; Andriole, 2005). Hetero-resistance, which is defined as the coexistence of drug-susceptible and drug-resistant isolates in clinical samples and may exhibit varying levels of drug susceptibility, appears to be more frequent in FQs resistance (Eilertson et al., 2014). The accuracy of WGS in predicting phenotypic resistance to rifampicin, isoniazid, and FQs is high in extensive comparative studies (Farhat et al., 2017; Meehan et al., 2019).

Understanding the evolution of FQs resistance in rifampicin-susceptible TB may help assess the effectiveness of TB programs and interventions and provide guidance for TB prevention and care in the future. However, little is known about FQs resistance in rifampicin-susceptible TB that emerged in China (Zhang et al., 2022). Zhejiang Province, as the first province in China to launch sentinel surveillance, has conducted drug resistance surveys following the initiation of the WHO/International Union Against Tuberculosis and Lung Disease global anti-TB drug resistance surveillance project in 1994. Zhejiang Province was also the first province in China to conduct periodic surveys for drug-resistant tuberculosis (DR-TB) since WHO integrated the country into its surveillance network for DR-TB in 1999.

To the best of our knowledge, our study is the first to address the varying prevalence patterns of FQs resistance among rifampicin-susceptible TB in a well-developed province based on the TB drug-resistance survey project. We provide the findings of a retrospective WGS analysis of all Mtb isolates collected from pulmonary TB cases in the Zhejiang area during TB drug resistance surveillance from 2018 to 2019. We aimed to investigate the prevalence of FQs resistance, especially among rifampicin-susceptible TB. Furthermore, we identified the instances of hetero-resistance in FQs-resistant strains and quantified the FQs resistance due to the transmission of FQs-resistant strains.

## Methods

### Study design and sample enrollment

The study was a retrospective study that included all the culture-positive patients diagnosed with TB at local TB dispensaries in Zhejiang Province during TB drug-resistance surveillance from Jan 1, 2018, to Dec 31, 2019. The Zhejiang ProvincialCenter for Disease Control and Prevention’s TB reference laboratory collected all clinical isolates from pulmonary TB patients for further species identification, strain preservation and WGS before each patient started anti-TB or other relevant clinical treatments. Records related to demographics, clinical and microbiology were retrieved from the national TB information management system.

### WGS and bioinformatics analysis

WGS was performed for each clinical isolate after it was collected. Mtb culture products were inactivated, and genomic DNA was extracted using a bacterial DNA extraction kit (QIAGEN Inc., Dusseldorf, Germany), following the manufacturer’s instructions. The purified genomic DNA was quantified using a TBS-380 fluorometer (Turner BioSystems Inc., Sunnyvale, CA, United States) to ensure compliance with quality requirements for library preparation, sequencing, and detection. Genomic DNA samples were treated and fragmented to approximately 400 bp. Sequencing libraries were generated using the NEXTflex^™^ Rapid DNA-Seq Kit, followed by multiplexing and loading onto the Illumina NovaSeq 6000 PE150 system (San Diego, CA92122, United States). Sequencing employed a 2 × 150 paired-end configuration. Raw sequencing data underwent processing with fastp v0.20.1 to remove adapter sequences and filter out low-quality bases (Chen et al., 2018). Subsequently, high-quality sequence data were then input into Kraken v1.1.1 for species identification. Samples identified as other species or with an Mtb proportion below 90% were rejected as contaminated (Wood and Salzberg, 2014). The remaining samples’ sequencing data were aligned to the H37Rv reference genome (NC_000962.3) using BWA v0.7.17 (Li, 2013). Samples meeting criteria with an average sequencing depth ≥ 20× and average genome coverage ≥95% were selected for subsequent data analysis. The SAMtools/BCFtools suite v1.13 facilitated the calling of fixed single-nucleotide polymorphisms (SNPs) (frequency ≥ 90%) at loci where the alternate alleles were supported by at least five reads (combining both forward and reverse reads) (Danecek et al., 2021).

### WGS-based DST

Clean sequencing data were input into the local version of TB-Profiler v4.4.2 and aligned to the reference genome of H37Rv to identify the genotype of resistance-associated mutations and detect the resistance profile of 9 anti-TB drugs. These drugs encompassed isoniazid, rifampicin, streptomycin, ethambutol, fluoroquinolones (levofloxacin and moxifloxacin), amikacin, kanamycin, and capreomycin (Phelan et al., 2019). Mutations with a frequency of less than 10% were excluded from the analysis. WGS-based DST results were deduced by assessing the presence or absence of mutations (Tier 1 and Tier 2 mutations) in a comprehensive database containing drug resistance-associated mutations, adhering to evidence levels recommended by WHO (Walker et al., 2022). In this study, hetero-resistance was defined based on the observation that the frequency of resistant alleles in the sequence reads was below 99%.

### Phylogeny construction and transmission cluster analysis

The fixed SNPs, excluding those located in proline-glutamic acid (PE)/proline-proline-glutamic acid (PPE) genes, insertion elements, repetitive regions, and genes associated with drug resistance, were combined into a concatenated alignment (Meehan et al., 2019). Maximum-likelihood (ML) phylogenetic trees were inferred from the concatenated alignment using IQ-Tree v2.2.5 (Nguyen et al., 2015). The best-scoring ML trees were rooted using *M. canettii* (RefSeq: NC_015848.1) as the outgroup and were visually represented with the Interactive Tree of Life (iTOL) (Letunic and Bork, 2021). A genomic threshold (≤12 SNPs) was applied to identify clusters of isolates potentially consistent with recent transmission referred to as genomic clusters (Yang et al., 2017). Primary FQs resistance (transmitted FQs resistance) was defined as the FQs resistance-conferring mutation shared by two or more strains within a genomic cluster. Any other FQs resistance-conferring mutations were categorized as acquired FQs resistance.

### Statistical analysis

Statistical analyses were conducted using the R package gtsummary (Sjoberg et al., 2021). The Pearson’s chi-squared or Fisher’s exact test was used for comparison of categorical variables, such as demographic, bacteriological, and clinical characteristics.

## Results

### Characteristics of the patients and Mtb isolates

**Figure 1:**
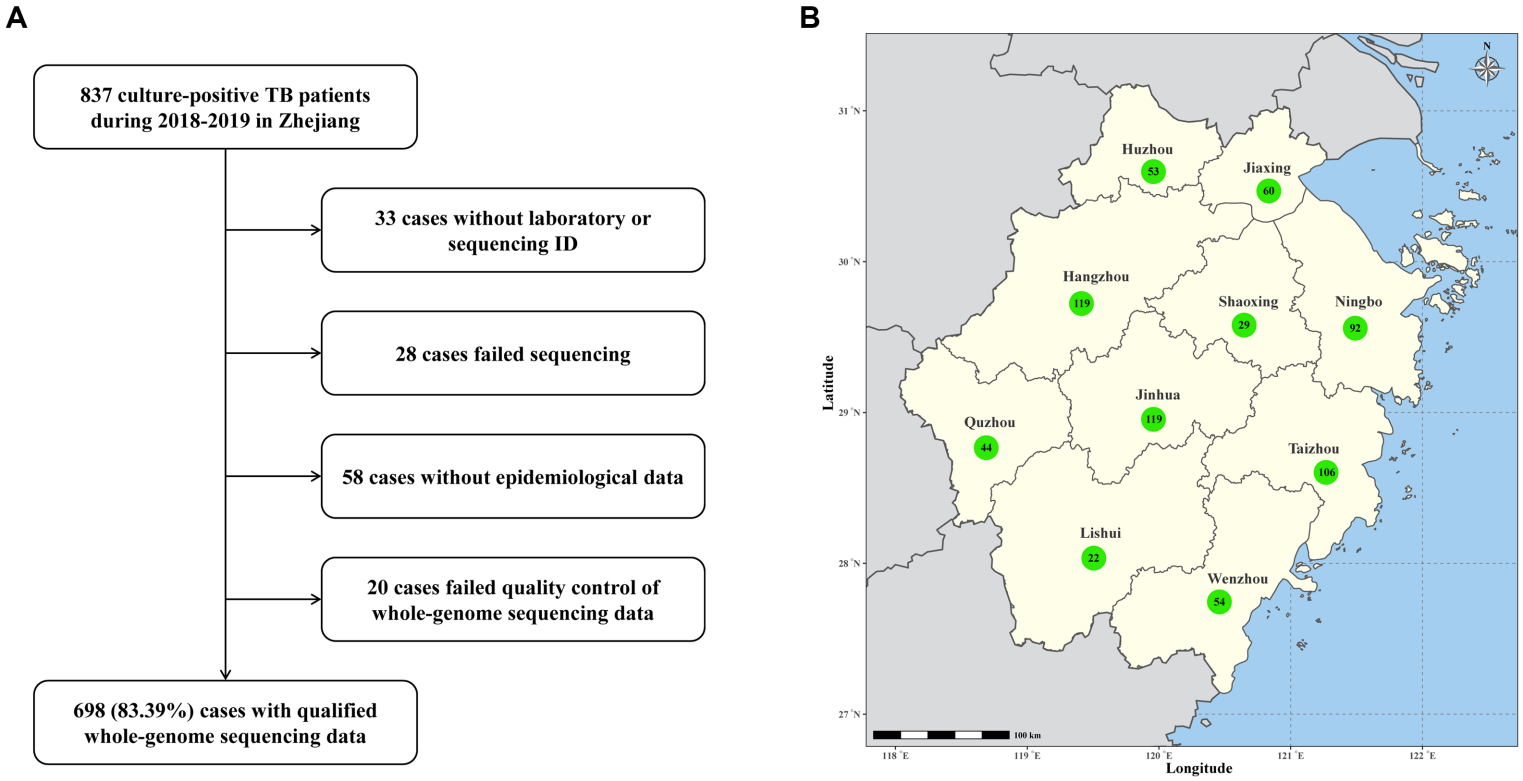
**(A)** Sample enrollment and study flowchart. **(B)** Map of Zhejiang Province with the number of TB cases from each prefecture-level city.

A total of 837 culture-positive patients diagnosed with TB in Zhejiang area during TB drug-resistance surveillance from January 1, 2018, to December 31, 2019 were enrolled in this study. Out of these patients, 139 (16.6%) were excluded from analysis due to strain contamination, recovery failure, WGS failure or loss of epidemiological data (Figure 1). The remaining 698 patients had an average age of 51 years (ranging from 13 to 91 years). 493 (70.6%) of them were male and 645 (92.4%) were new TB cases. Based on the results of WGS-based DST, 22 (3.2%) were diagnosed with RR-TB. Notably, RR-TB patients were relatively younger, exhibiting a statistically significant difference compared to rifampicin-susceptible patients (median age 62 years vs. 67.5 years, *p* = 0.009). Further analysis revealed RR-TB patients were more likely to be migrant (59% vs. 38%, *p* = 0.049) and have been previously treated for TB (22.7% vs. 7.1%; *p* = 0.020) than rifampicin-susceptible TB patients (Table 1).

**Table 1:** Demographic, clinical, and bacteriological characteristics of RR-TB and rifampicin-susceptible TB cases in Zhejiang Province.

			Rifampicin		
	*N*	Overall, *n* = 698^a^	Resistant, *n* = 22^a^	Sensitive, *n* = 676^a^	*p*-value^b^
**Age**	698				**0.009**
≤25		111 (16%)	4 (18%)	107 (16%)	
25~		180 (26%)	11 (50%)	169 (25%)	
45~		198 (28%)	6 (27%)	192 (28%)	
65~		209 (30%)	1 (4.5%)	208 (31%)	
**Gender**	698				0.5
Male		493 (71%)	17 (77%)	476 (70%)	
Female		205 (29%)	5 (23%)	200 (30%)	
**Census register**	698				**0.049**
Resident		426 (61%)	9 (41%)	417 (62%)	
Migrant		272 (39%)	13 (59%)	259 (38%)	
**TB treatment history**	698				**0.020**
No		645 (92%)	17 (77%)	628 (93%)	
Yes		53 (7.6%)	5 (23%)	48 (7.1%)	
**Diabetes mellitus**	685				0.7
No		602 (88%)	19 (86%)	583 (88%)	
Yes		83 (12%)	3 (14%)	80 (12%)	
**Hepatitis B**	679				0.12
No		660 (97%)	20 (91%)	640 (97%)	
Yes		19 (2.8%)	2 (9.1%)	17 (2.6%)	
**Cluster**^c^	698				0.4
Unique		648 (93%)	22 (100%)	626 (93%)	
Cluster		50 (7.2%)	0 (0%)	50 (7.4%)	
**Lineage**	698				0.12
Non-Beijing		197 (28%)	3 (14%)	194 (29%)	
Ancient Beijing		27 (3.9%)	2 (9.1%)	25 (3.7%)	
Modern Beijing		474 (68%)	17 (77%)	457 (68%)	

a *n* (%).

b Pearson’s Chi-squared test, Fisher’s exact test (Bold values denote statistical significance at the *P*-value < 0.05 level).

c Genomic cluster identified by a threshold (≤12 SNPs).

### Prevalence and mutation types of FQs resistance

According to the results of WGS-based DST, 32 (4.6%) exhibited resistance to FQs, including 27 rifampicin-susceptible isolates and 5 rifampicin-resistant isolates. The rate of FQs resistance was 4.0% (27/676) among rifampicin-susceptible TB and 22.7% (5/22) among RR-TB. The drug resistance profile and epidemiological information of 32 FQs -resistant TB patients were shown in Supplementary Table S1.

Five rifampicin-resistant isolates harbored the FQs resistance-conferring mutations in the *gyrA* and *gyrB* genes, including two *gyrA* D94A, two *gyrA* A90V and one *gyrB* E501D. Among 676 rifampicin-susceptible isolates, 27 harbored diverse FQs resistance-conferring mutations, including ten *gyrA* D94A, seven *gyrA* A90V, six *gyrA* D94N, one *gyrA* D94Y, one *gyrB* A504V, and one *gyrB* D461N. Additionally, one rifampicin-susceptible isolates harbored more than one FQs resistance-conferring mutation, *gyrA* A90V + *gyrA* S91P.

**Figure 2:**
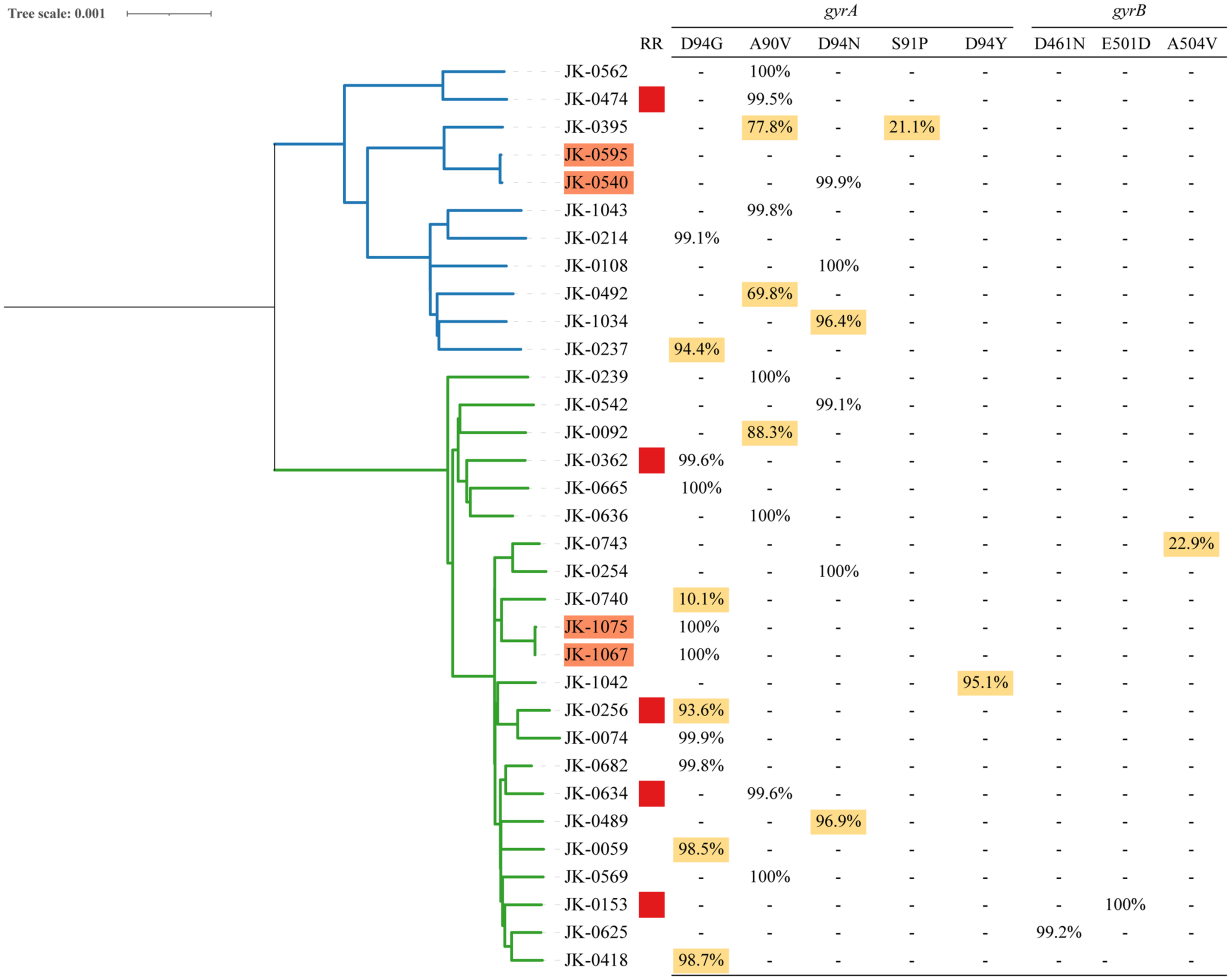
Phylogeny, clustering and FQs hetero-resistance profile of 32 FQs-resistant Mtb isolates. (1) Blue and green branches indicate non-Beijing and Beijing strains, respectively. (2) Genomic-clustered strains differing by ≤12 SNPs are highlighted in orange. (3) Red square indicates the Mtb isolate resistant to rifampicin. (4) Allele frequency of FQs resistance-conferring loci in *gyrA* and *gyrB* is shown in the right side. Hetero-resistance mutations are highlighted in yellow.

Among 27 rifampicin-susceptible isolates with FQs resistance-conferring mutations, 11 (40.7%) were identified as FQ hetero-resistance. The allele frequencies of FQ hetero-resistance ranged from 10.1 to 98.7%. Regarding FQs hetero-resistant isolates, 26 had a single unfixed mutation and one had multiple unfixed mutations in *gyrA*. FQs hetero-resistance was only observed in one isolate among 5 rifampicin-resistant isolates (20%, 1/5). The allele frequencies of FQs resistance-conferring mutations in 32 FQs-resistant isolates were shown in Figure 2.

### Transmission of FQ-resistant isolates

All 698 isolates were divided into 24 clusters defined as strains that differed by 12 or fewer SNPs, among which two clusters included FQs-resistant isolates. There are two basic ways that drug resistance emerges: acquired drug resistance arising from insufficient therapy or primary drug resistance as a result of the transmission of drug-resistant strains. Notably, we were able to observe primary and acquired drug resistance in each of the clusters with FQs-resistant isolates, separately, as depicted in Figure 2. Within one cluster (JK-1075 and JK-1067), we identified a uniform fixed mutation conferring FQsresistance (*gyrA* D94G) present in both isolates, with a 100% allele frequency. This observation strongly suggests primary drug resistance resulting from transmission. In another cluster (JK-0595 and JK-0540), acquired drug resistance was evident, where only one of the two isolates exhibited FQs resistance, featuring a fixed mutation conferring resistance (*gyrA* D94N, 99.88%).

## Discussion

Rapid, reliable, and increasingly affordable WGS technology can help with TB prevention and care, including diagnosis, treatment, and surveillance (Satta et al., 2018; van der Werf and Ködmön, 2019). It has demonstrated efficacy in discerning genetic drug resistance profiles (Jabbar et al., 2019; Wu et al., 2022). In this study, we have investigated the prevalence of FQs resistance and its transmission in all Mtb isolates from pulmonary TB patients during TB drug-resistance surveillance in Zhejiang Province. Our findings reveal that the majority (84.4%, 27/32) of FQs resistant TB were susceptible to rifampicin, with a notable proportion (40.7%, 11/27) identified as hetero-resistance. WGS data indicate that the transmission of FQs-resistant strains contributed to 3.7% (1/27) of FQs resistance in rifampicin-susceptible TB.

We observed that more than two-thirds of the FQs-resistant cases were identified in rifampicin-susceptible TB patients. Regard as the core agents for RR-TB treatment, moxifloxacin and levofloxacin are usually prescribed to RR-TB patients (Kayalı et al., 2021; World Health Organization, 2022a). This prescription practice aligns with the technical specifications on TB prevention and care. Surveillance data on the prevalence of FQs resistance in rifampicin-susceptible TB are scarce, and the DST of FQs is not routinely conducted for rifampicin-susceptible TB patients (Heyckendorf et al., 2018; Tagliani et al., 2021). Previous review studies showed that prevalence of FQs resistance in Mtb clinical isolates in Shanghai was higher (6.1%) than reported in other countries, where the range is typically 0 to 4.4% (Ginsburg et al., 2003; Ho et al., 2014; Kayalı et al., 2021; Schwalb et al., 2021; Zhang et al., 2022). FQs are also the most often recommended antibiotics in China for respiratory infections. Therefore, it is imperative to conduct surveillance on FQs resistance and monitor fluoroquinolones exposure in newly diagnosed TB patients in countries with a high TB burden.

Previous studies have indicated that mutations in the quinolones resistance determining region of *gyrA* or *gyrB* constitute the main mechanism for Mtb resistance to fluoroquinolones (Gröschel et al., 2021; Maruri et al., 2021). Within the scope of this study, the majority of FQs resistance-conferring mutations occurring in rifampicin-resistant Mtb were D94G (2/5) or A90V (2/5). Our findings suggest that Mtb strains harboring multiple other drug resistance-conferring mutations might be more likely to acquire FQs resistance-conferring mutations with a lower fitness cost. The profile of FQs resistance-conferring mutations occurring in rifampicin-susceptible Mtb exhibited greater diversity. Every instance of FQs hetero-resistance was observed in rifampicin-susceptible Mtb, with the exception of a single rifampicin-resistant Mtb isolate. Hetero-resistance represents a critical stage in the development of an initially drug-susceptible Mtb population toward complete resistance to a specific drug over the course of an infection (Dheda et al., 2018). Non-lethal drug concentrations facilitate the emergence and selection of drug mutations with a low fitness cost (Castro et al., 2020). According to the hypotheses, the characteristics of FQs resistance-conferring mutations in rifampicin-susceptible Mtb imply that the Mtb FQs resistance may have been induced by ineffective FQs treatment. Due to easy accessibility and improper use of FQs, several studies have indicated a high proportion of TB patients being exposed to FQs before their TB diagnosis (Yang et al., 2015; Yuen et al., 2015). Moreover, hetero-resistance hampers the effectiveness of rapid molecular assays in detecting drug resistance (O’Donnell et al., 2019). Utilizing WGS techniques, variant allele frequencies could be employed to identify hetero-resistance for drug resistance prediction (Chaidir et al., 2019). In our study, we identified FQs hetero-resistance in rifampicin-susceptible Mtb isolates, with hetero-resistance frequencies ranging from 10.1 to 98.7%. Our findings revealed that WGS is sensitive in detecting FQs resistance in hetero-resistant strains and the real burden of FQs resistance in rifampicin-susceptible TB might be underestimated.

Previous studies have demonstrated that the transmission of rifampicin-resistant strains facilitated the spread of FQs resistance (Takiff and Feo, 2015; Nikolayevskyy et al., 2019). In the present study, FQs resistance caused by transmission was also observed in rifampicin-susceptible Mtb isolates within the Zhejiang region. Comparative analysis of transmission and resistance-conferring mutations in FQs-resistant isolates revealed a minimal incidence of FQs resistance in rifampicin-susceptible isolates (3.7%, 1/27) and no occurrences in rifampicin-resistant isolates (0/5) resulting from the transmission of FQs-resistant strains. The higher prevalence of FQs resistance in rifampicin-susceptible TB cases, as compared to rifampin-resistant TB cases, is likely due to the wide use of FQs for antibiotic therapy throughout Zhejiang. Moreover, the empirical prescription of FQs for respiratory infections has been linked to delays in the detection and treatment of pulmonary tuberculosis. These delays may, in turn, contribute to the increased transmission of TB (Lin et al., 2016).

The retrospective study design of our study limited the access to information regarding FQs prescriptions for TB patients prior to diagnosis, including details such as prescription date, dosage, specific type of FQs, and duration of supply. However, to the best of our knowledge, this study represents the first investigation into the prevalence of FQs resistance among rifampicin-susceptible TB cases in Zhejiang Province (Zhang et al., 2022). Analysis of WGS data revealed that 50% of FQs resistance in rifampicin-susceptible TB cases manifested as hetero-resistance, and the transmission of FQs -resistant strains contributed to the development of FQ-resistant TB. In order to mitigate the emergence of FQ resistance, it is imperative to promptly establish a WGS-based surveillance system for TB. Additionally, raising clinical awareness regarding the judicious use of FQs for respiratory infections is essential.

## Conclusion

The prevalence of FQs resistance among TB patients who were rifampicin-susceptible was severein Zhejiang. The emergence of FQs resistance in TB isolates susceptible to rifampicin mainly stems from the selection of drug-resistant strains. In order to prevent the development of FQs resistance, the WGS-based surveillance system for TB should be urgently established, and clinical awareness of the responsible use of FQs for respiratory infections should be enhanced.

## Data availability statement

The datasets presented in this study can be found in online repositories. The names of the repository/repositories and accession number(s) can be found at: https://ngdc.cncb.ac.cn/bioproject/browse/PRJCA018552.

## Ethics statement

This study was approved by the Ethics Committee of the Zhejiang Provincial Center for Disease Control and Prevention. All eligible participants who agreed to participate in the program and signed an informed consent form were required to complete a questionnaire and provide at least one sputum specimen for subsequent studies.

## Author contributions

YC: Writing – review & editing, Writing – original draft, Methodology, Funding acquisition, Formal analysis, Conceptualization. YL: Writing – review & editing, Writing – original draft, Formal analysis, Data curation, Conceptualization. YZ: Writing – review & editing, Formal analysis, Data curation. TH: Writing – review & editing, Formal analysis, Data curation. XL: Writing – review & editing, Formal analysis, Data curation. JunlG: Writing – review & editing, Data curation. JunsG: Writing – review & editing, Data curation. XW: Writing – review & editing, Formal analysis, Data curation. ZL: Writing – review & editing, Funding acquisition, Data curation. FT: Writing – review & editing, Data curation.

## References

[ref1] Andriole V. T. (2005). The quinolones: past, present, and future. Clin. Infect. Dis. 41, S113–S119. doi: 10.1086/428051, PMID: 15942877

[ref2] Castro R. A. Ross A. Kamwela L. Reinhard M. Loiseau C. Feldmann J.. (2020). The genetic background modulates the evolution of fluoroquinolones-resistance in *Mycobacterium tuberculosis*. Mol. Biol. Evol. 37, 195–207. doi: 10.1093/molbev/msz214, PMID: 31532481 PMC6984360

[ref3] Chaidir L. Ruesen C. Dutilh B. E. Ganiem A. R. Andryani A. Apriani L.. (2019). Use of whole-genome sequencing to predict *Mycobacterium tuberculosis* drug resistance in Indonesia. J. Glob. Antimicrob. Resist. 16, 170–177. doi: 10.1016/j.jgar.2018.08.018, PMID: 30172045

[ref5] Chen S. Zhou Y. Chen Y. Gu J. (2018). Fastp: an ultra-fast all-in-one FASTQ preprocessor. Bioinformatics 34, i884–i890. doi: 10.1093/bioinformatics/bty560 30423086 PMC6129281

[ref6] Cohen K. A. Manson A. L. Desjardins C. A. Abeel T. Earl A. M. (2019). Deciphering drug resistance in *Mycobacterium tuberculosis* using whole-genome sequencing: progress, promise, and challenges. Genome Med. 11:45. doi: 10.1186/s13073-019-0660-8, PMID: 31345251 PMC6657377

[ref7] Danecek P. Bonfield J. K. Liddle J. Marshall J. Ohan V. Pollard M. O.. (2021). Twelve years of SAMtools and BCFtools. Gigascience 10:giab008. doi: 10.1093/gigascience/giab008, PMID: 33590861 PMC7931819

[ref9] Dheda K. Lenders L. Magombedze G. Srivastava S. Raj P. Arning E.. (2018). Drug-penetration gradients associated with acquired drug resistance in patients with tuberculosis. Am. J. Respir. Crit. Care Med. 198, 1208–1219. doi: 10.1164/rccm.201711-2333OC, PMID: 29877726 PMC6221573

[ref10] Dorman S. E. Nahid P. Kurbatova E. V. Phillips P. P. J. Bryant K. Dooley K. E.. (2021). Four-month Rifapentine regimens with or without moxifloxacin for tuberculosis. N. Engl. J. Med. 384, 1705–1718. doi: 10.1056/NEJMoa2033400 33951360 PMC8282329

[ref11] Eilertson B. Maruri F. Blackman A. Herrera M. Samuels D. C. Sterling T. R. (2014). High proportion of Heteroresistance in *gyrA* and *gyrB* in fluoroquinolones-resistant *Mycobacterium tuberculosis* clinical isolates. Antimicrob. Agents Chemother. 58, 3270–3275. doi: 10.1128/AAC.02066-13, PMID: 24687490 PMC4068501

[ref12] Farhat M. R. Jacobson K. R. Franke M. F. Kaur D. Murray M. Mitnick C. D. (2017). Fluoroquinolones resistance mutation detection is equivalent to culture-based drug sensitivity testing for predicting multidrug-resistant tuberculosis treatment outcome: a retrospective cohort study. Clin. Infect. Dis. 65, 1364–1370. doi: 10.1093/cid/cix556, PMID: 29017248 PMC5850426

[ref13] Ginsburg A. S. Hooper N. Parrish N. Dooley K. E. Dorman S. E. Booth J.. (2003). Fluoroquinolones resistance in patients with newly diagnosed tuberculosis. Clin. Infect. Dis. 37, 1448–1452. doi: 10.1086/379328 14614666

[ref14] Gröschel M. I. Owens M. Freschi L. Vargas R. Marin M. G. Phelan J.. (2021). GenTB: a user-friendly genome-based predictor for tuberculosis resistance powered by machine learning. Bioinformatics. 13, 138. doi: 10.1101/2021.03.27.437319 PMC8407037 34461978

[ref15] Heyckendorf J. Andres S. Köser C. U. Olaru I. D. Schön T. Sturegård E.. (2018). What is resistance? Impact of phenotypic versus molecular drug resistance testing on therapy for multi- and extensively drug-resistant tuberculosis. Antimicrob. Agents Chemother. 62, e01550–e01517. doi: 10.1128/AAC.01550-17, PMID: 29133554 PMC5786814

[ref16] Ho J. Jelfs P. Sintchenko V. (2014). Fluoroquinolones resistance in non-multidrug-resistant tuberculosis—a surveillance study in New South Wales, Australia, and a review of global resistance rates. Int. J. Infect. Dis. 26, 149–153. doi: 10.1016/j.ijid.2014.03.1388, PMID: 25086437

[ref17] Hu Y. Mathema B. Wang W. Kreiswirth B. Jiang W. Xu B. (2011). Population-based investigation of fluoroquinoloness resistant tuberculosis in rural eastern China. Tuberculosis 91, 238–243. doi: 10.1016/j.tube.2011.03.001, PMID: 21450523

[ref18] Jabbar A. Phelan J. E. de Sessions P. F. Khan T. A. Rahman H. Khan S. N.. (2019). Whole genome sequencing of drug resistant *Mycobacterium tuberculosis* isolates from a high burden tuberculosis region of north West Pakistan. Sci. Rep. 9:14996. doi: 10.1038/s41598-019-51562-6, PMID: 31628383 PMC6802378

[ref19] Kayalı R. A. Özkan S. A. Biçmen C. Erer OF (2021). The relation between the emergence of fluoroquinolones resistance and fluoroquinolones exposure in new cases of active pulmonary tuberculosis. Turk. Thorac. J. 22:2002272. doi: 10.5152/TurkThoracJ.2021.19128 PMC7919434 33646103

[ref20] Letunic I. Bork P. (2021). Interactive tree of life (iTOL) v5: an online tool for phylogenetic tree display and annotation. Nucleic Acids Res. 49, W293–W296. doi: 10.1093/nar/gkab301, PMID: 33885785 PMC8265157

[ref21] Li H. (2013). Aligning sequence reads, clone sequences and assembly contigs with BWA-MEM. arXiv. doi: 10.48550/arXiv.1303.3997

[ref22] Lin H. Dyar O. J. Rosales-Klintz S. Zhang J. Tomson G. Hao M.. (2016). Trends and patterns of antibiotic consumption in Shanghai municipality, China: a 6 year surveillance with sales records, 2009–14. J. Antimicrob. Chemother. 71, 1723–1729. doi: 10.1093/jac/dkw013, PMID: 26892776

[ref23] Maruri F. Guo Y. Blackman A. van der Heijden Y. F. Rebeiro P. F. Sterling T. R. (2021). Resistance-conferring mutations on whole-genome sequencing of fluoroquinolones-resistant and-susceptible *Mycobacterium tuberculosis* isolates: a proposed threshold for identifying resistance. Clin. Infect. Dis. 72, 1910–1918. doi: 10.1093/cid/ciaa496, PMID: 32348473 PMC8315129

[ref24] Meehan C. J. Goig G. A. Kohl T. A. Verboven L. Dippenaar A. Ezewudo M.. (2019). Whole genome sequencing of *Mycobacterium tuberculosis*: current standards and open issues. Nat. Rev. Microbiol. 17, 533–545. doi: 10.1038/s41579-019-0214-5, PMID: 31209399

[ref25] Miotto P. Tessema B. Tagliani E. Chindelevitch L. Starks A. M. Emerson C.. (2017). A standardised method for interpreting the association between mutations and phenotypic drug resistance in *Mycobacterium tuberculosis*. Eur. Respir. J. 50:1701354. doi: 10.1183/13993003.01354-2017 29284687 PMC5898944

[ref26] Nguyen L.-T. Schmidt H. A. Von Haeseler A. Minh B. Q. (2015). IQ-TREE: a fast and effective stochastic algorithm for estimating maximum-likelihood phylogenies. Mol. Biol. Evol. 32, 268–274. doi: 10.1093/molbev/msu300, PMID: 25371430 PMC4271533

[ref27] Nikolayevskyy V. Niemann S. Anthony R. Van Soolingen D. Tagliani E. Ködmön C.. (2019). Role and value of whole genome sequencing in studying tuberculosis transmission. Clin. Microbiol. Infect. 25, 1377–1382. doi: 10.1016/j.cmi.2019.03.022, PMID: 30980928

[ref28] O’Donnell M. R. Larsen M. H. Brown T. S. Jain P. Munsamy V. Wolf A.. (2019). Early detection of emergent extensively drug-resistant tuberculosis by flow cytometry-based phenotyping and whole-genome sequencing. Antimicrob. Agents Chemother. 63, e01834–e01818. doi: 10.1128/AAC.01834-18, PMID: 30670422 PMC6437479

[ref29] Phelan J. E. O’Sullivan D. M. Machado D. Ramos J. Oppong Y. E. A. Campino S.. (2019). Integrating informatics tools and portable sequencing technology for rapid detection of resistance to anti-tuberculous drugs. Genome Med. 11:41. doi: 10.1186/s13073-019-0650-x, PMID: 31234910 PMC6591855

[ref30] Ruiz J. (2003). Mechanisms of resistance to quinolones: target alterations, decreased accumulation and DNA gyrase protection. J. Antimicrob. Chemother. 51, 1109–1117. doi: 10.1093/jac/dkg222, PMID: 12697644

[ref31] Satta G. Lipman M. Smith G. P. Arnold C. Kon O. M. McHugh T. D. (2018). Mycobacterium tuberculosis and whole-genome sequencing: how close are we to unleashing its full potential? Clin. Microbiol. Infect. 24, 604–609. doi: 10.1016/j.cmi.2017.10.030, PMID: 29108952

[ref32] Schwalb A. Cachay R. Meza E. Cáceres T. Blackman A. Maruri F.. (2021). Fluoroquinolones susceptibility in first-line drug-susceptible *M. tuberculosis* isolates in Lima, Peru. BMC. Res. Notes 14:413. doi: 10.1186/s13104-021-05832-0, PMID: 34776013 PMC8591909

[ref33] Siddiqui S. Brooks M. B. Malik A. A. Fuad J. Nazish A. Bano S.. (2019). Evaluation of GenoType MTBDR plus for the detection of drug-resistant *Mycobacterium tuberculosis* on isolates from Karachi, Pakistan. PLoS ONE 14:e0221485. doi: 10.1371/journal.pone.0221485, PMID: 31425565 PMC6699735

[ref34] Sjoberg D. D. Whiting K. Curry M. Lavery J. A. Larmarange J. (2021). Reproducible summary tables with the gtsummary package. R Journal 13, 570–580. doi: 10.32614/RJ-2021-053

[ref35] Tagliani E. Anthony R. Kohl T. A. De Neeling A. Nikolayevskyy V. Ködmön C.. (2021). Use of a whole genome sequencing-based approach for *Mycobacterium tuberculosis* surveillance in Europe in 2017–2019: an ECDC pilot study. Eur. Respir. J.:57. doi: 10.1183/13993003.02272-2020 PMC7784142 32732329

[ref36] Takiff H. E. Feo O. (2015). Clinical value of whole-genome sequencing of *Mycobacterium tuberculosis*. Lancet Infect. Dis. 15, 1077–1090. doi: 10.1016/S1473-3099(15)00071-7 26277037

[ref37] van der Werf M. J. Ködmön C. (2019). Whole-genome sequencing as tool for investigating international tuberculosis outbreaks: a systematic review. Front. Public Health 7:87. doi: 10.3389/fpubh.2019.00087 31058125 PMC6478655

[ref38] Walker T. M. Merker M. Kohl T. A. Crook D. W. Niemann S. Peto T. E. A. (2017). Whole genome sequencing for M/XDR tuberculosis surveillance and for resistance testing. Clin. Microbiol. Infect. 23, 161–166. doi: 10.1016/j.cmi.2016.10.014 27789378

[ref39] Walker T. M. Miotto P. Köser C. U. Fowler P. W. Knaggs J. Iqbal Z.. (2022). The 2021 WHO catalogue of *Mycobacterium tuberculosis* complex mutations associated with drug resistance: a genotypic analysis. Lancet Microbe 3, e265–e273. doi: 10.1016/S2666-5247(21)00301-3, PMID: 35373160 PMC7612554

[ref40] Wang J.-Y. Hsueh P.-R. Jan I.-S. Lee L.-N. Liaw Y.-S. Yang P.-C.. (2006). Empirical treatment with a fluoroquinolones delays the treatment for tuberculosis and is associated with a poor prognosis in endemic areas. Thorax 61, 903–908. doi: 10.1136/thx.2005.056887, PMID: 16809417 PMC2104756

[ref41] Witney A. A. Cosgrove C. A. Arnold A. Hinds J. Stoker N. G. Butcher P. D. (2016). Clinical use of whole genome sequencing for *Mycobacterium tuberculosis*. BMC Med. 14:46. doi: 10.1186/s12916-016-0598-2, PMID: 27004841 PMC4804576

[ref42] Wood D. E. Salzberg S. L. (2014). Kraken: ultrafast metagenomic sequence classification using exact alignments. Genome Biol. 15, 1–12. doi: 10.1186/gb-2014-15-3-r46 PMC4053813 24580807

[ref43] World Health Organization (2020). WHO consolidated guidelines on tuberculosis. Module 4: treatment-drug-resistant tuberculosis treatment, Geneva: World Health Organization. 32603040

[ref44] World Health Organization (2022a). WHO operational handbook on tuberculosis. Module 4: treatment-drug-resistant tuberculosis treatment, 2022 update, Geneva: World Health Organization.

[ref45] World Health Organization (2022b). WHO consolidated guidelines on tuberculosis. Module 4: treatment-drug-susceptible tuberculosis treatment, Geneva: World Health Organization. 35727905

[ref46] Wu X. Tan G. Sha W. Liu H. Yang J. Guo Y.. (2022). Use of whole-genome sequencing to predict *Mycobacterium tuberculosis* complex drug resistance from early positive liquid cultures. Microbiol. Spectr. 10, e02516–e02521. doi: 10.1128/spectrum.02516-21, PMID: 35311541 PMC9045259

[ref47] Yang C. Luo T. Shen X. Wu J. Gan M. Xu P.. (2017). Transmission of multidrug-resistant *Mycobacterium tuberculosis* in Shanghai, China: a retrospective observational study using whole-genome sequencing and epidemiological investigation. Lancet Infect. Dis. 17, 275–284. doi: 10.1016/S1473-3099(16)30418-2, PMID: 27919643 PMC5330813

[ref48] Yang C. Shen X. Peng Y. Lan R. Zhao Y. Long B.. (2015). Transmission of *Mycobacterium tuberculosis* in China: a population-based molecular epidemiologic study. Clin. Infect. Dis. 61, 219–227. doi: 10.1093/cid/civ255, PMID: 25829000 PMC4490233

[ref49] Yuen C. M. Amanullah F. Dharmadhikari A. Nardell E. A. Seddon J. A. Vasilyeva I.. (2015). Turning off the tap: stopping tuberculosis transmission through active case-finding and prompt effective treatment. Lancet 386, 2334–2343. doi: 10.1016/S0140-6736(15)00322-0, PMID: 26515675 PMC7138065

[ref50] Zhang Y. Jiang Y. Yu C. Li J. Shen X. Pan Q.. (2022). Whole-genome sequencing for surveillance of fluoroquinolone resistance in rifampicin-susceptible tuberculosis in a rural district of Shanghai: a 10-year retrospective study. Front. Public Health 10:990894. doi: 10.3389/fpubh.2022.990894 36187694 PMC9521709

